# Annotations

**Published:** 1900-05-26

**Authors:** 


### Annotations.

### Brandy.

Sophistication has become so much the order of the day that the price of some things seems to be fixed, not by that of the real article, but by that of some reasonably good substitute. So only can we explain the action of the Contracts Committee of the Metropolitan Asylums Board in proposing to supply its hospitals with "brandy" at 11s. 6d. a gallon. Probably they thought that brandy was a fancy article, the nature and origin of which they could in no case be sure about, and that if they did but get a pure spirit they would be doing the best under the circumstances. Still, if a patient requires brandy, brandy he should have, and not some grain-spirit substitute. We have, then, every sympathy with the resolution proposed by Professor W. R. Smith at the last meeting of the Board, that the proposal to buy "brandy" at 11s. 6d. a gallon should be referred back to the Contracts Committee, whence it emanated, for reconsideration. We will not enter here into the chemistry of the matter. Suffice it to say that very different ethers enter into the composition of the various kinds of spirit—brandy, whisky, &c.—and that, as everyone knows, their effects upon the system are essentially different. As Professor Smith said, brandy, so far as its use in hospitals is concerned, is in every sense a drug, and should, like other drugs, be pure, and up to the standard demanded by the British Pharmacopœia, in which brandy is defined as "a spirituous liquid distilled from wine and matured by age." Since the phylloxera invaded France, the amount of brandy obtained from grapes has so diminished in quantity that it has not been sufficient to meet the demand for it, and the deficiency has been made up by grain-spirit, which, although it can be flavoured to taste very like the real thing, is not brandy. After some discussion, Professor Smith's proposal was agreed to. We cannot see why there should have been any discussion about it. If the Board chooses to decide that grain-spirit is the only form in which spirituous liquors are to be administered to its patients, well and good. Let them have good whisky and have done with it. Or if the Board takes up the position that it is for the sake of the alcohol alone that brandy is given, let us order absolute alcohol watered down to the proper strength, as Sir Benjamin Richardson used to do. But in heaven's name let us have no shams. So long as brandy is retained on the list of extras which may be ordered, the brandy which is given should be real and pure.

### Mecca and its Pilgrimage.

It might be imagined that with the extension on every hand of railways, telegraphs, and all the conveniences which go to make up modern civilisation old faiths would die, old superstitions would fade away, and many of the more uncomfortable of religious exercises would fall into disuse. Such, however, is far from being the case. Some of the most uncompromising religions have turned to their own purposes the inventions and devices of iconoclastic science, and now we find it announced that the Sultan has authorised the construction of a railway to Mecca! This is a suggestion which will not arouse any special feeling of gratification among Europeans. From the Mohammedan point of view there is everything to be said in its favour. Mohammedans are taught that it is their duty to make the pilgrimage to the Tomb of the Prophet, and so great are the sufferings and the risks to life involved in this pilgrimage, that we cannot be surprised at the more intelligent among them urging any means which shall tend to lessen its dangers and to diminish the intolerable blood-tax on their co-religionists which it involves. But to the outside world the matter is very different. The congregation of great masses of people from all parts of the globe amid the conditions which obtain in the Hedjaz during the time of the pilgrimage is a constant sanitary danger to Europe. This danger has, however, been kept in check by the slowness of the pilgrims' progress. In the ancient days many died on the road. Whole shiploads of pilgrims with their crews perished from plague or cholera or other fatal diseases, and in the same way whole caravans left their bones to whiten on the desert sands; while such as escaped spent weary months in their journeyings, and so cleared themselves of infection. But with the advent of steamships the strictness of this natural quarantine, the efficacy of the *cordon sanitaire* resulting from long sea voyages and journeys on the desert, was much diminished, and the danger to the outside world arising from this most insanitary assemblage was proportionately increased. Many conferences of the nations have been held with the object of lessening the risks to which Europe has been exposed by the increased rapidity of this pilgrim traffic, and to a large extent the measures taken have proved effectual. If, however, a railway to Mecca were to come into existe nce the problem would assume quite a new feature, for although to the Mohammedans the pilgrimage would be made easy, to the outer world the danger would be increased.

### Rotten Fruit.

A very important case has been decided by the Court of Queen's Bench, in regard to a question raised at the Thames Police-Court last summer, as to the interpretation of a clause in the Public Health (London) Act, under which the destruction of unsound fruit and other articles is effected. The question was whether before a magistrate, acting under section 47 of this Act, condemns an article as being unsound, or unwholesome, or unfit for the food of man, he must have before him evidence that the goods were intended for the food of man. The case in point was one in which an application was made to a magistrate to condemn 117 tubs of strawberries which the inspector saw in a van in the street. It was admitted that the strawberries were unfit for the food of man, but, however obvious their destination might seem, no evidence was given that the fruit was, in fact, intended for the food of man, or was sold, or exposed for sale, or deposited for the purposes of sale, at the time of seizure; moreover, the consignee had refused to receive them. The magistrate refused to convict. According to the judgment now given, however, the strawberries ought to have been condemned, and it is laid down that so far as an order for the destruction of such articles is concerned, the question for the magistrate to decide is merely whether the goods are, in fact, unsound, unwholesome, or unfit for the food of man. It is otherwise, however, where the person exposing is charged on a summons for an offence under the Act.

